# The central proline rich region of POB1/REPS2 plays a regulatory role in epidermal growth factor receptor endocytosis by binding to 14-3-3 and SH3 domain-containing proteins

**DOI:** 10.1186/1471-2091-9-21

**Published:** 2008-07-22

**Authors:** Laura Tomassi, Anna Costantini, Salvatore Corallino, Elena Santonico, Martina Carducci, Gianni Cesareni, Luisa Castagnoli

**Affiliations:** 1 Department of Biology, University of Rome Tor Vergata, Via della Ricerca Scientifica, 00133 Rome, Italy; 2 IRCCS Fondazione Santa Lucia, 00143 Rome, Italy

## Abstract

**Background:**

The human POB1/REPS2 (Partner of RalBP1) protein is highly conserved in mammals where it has been suggested to function as a molecular scaffold recruiting proteins involved in vesicular traffic and linking them to the actin cytoskeleton remodeling machinery. More recently POB1/REPS2 was found highly expressed in androgen-dependent prostate cancer cell lines, while one of its isoforms (isoform 2) is down regulated during prostate cancer progression.

**Results:**

In this report we characterize the central proline rich domain of POB1/REPS2 and we describe for the first time its functional role in receptor endocytosis. We show that the ectopic expression of this domain has a dominant negative effect on the endocytosis of activated epidermal growth factor receptor (EGFR) while leaving transferrin receptor endocytosis unaffected. By a combination of different approaches (phage display, bioinformatics predictions, peptide arrays, mutagenic analysis, in vivo co-immunoprecipitation), we have identified two closely spaced binding motifs for 14-3-3 and for the SH3 of the proteins Amphiphysin II and Grb2. Differently from wild type, proline rich domains that are altered in these motifs do not inhibit EGFR endocytosis, suggesting that these binding motifs play a functional role in this process.

**Conclusion:**

Our findings are relevant to the characterization of the molecular mechanism underlying the involvement of POB1/REPS2, SH3 and 14-3-3 proteins in receptor endocytosis, suggesting that 14-3-3 could work by bridging the EGF receptor and the scaffold protein POB1/REPS2.

## Background

POB1/REPS2 (from now on POB1) was initially identified in a yeast two-hybrid screening as a partner of RalBP1 [1], an effector of the small G protein Ral [2] that displays a GAP activity towards Rac1 and Cdc42 [3]. POB1 is expressed as two isoforms, the short isoform is 521 residues long while the second one differs due to its having a 139 amino acid extension at the amino-terminus. The most prominent structural/functional features, which are common to both isoforms, include an amino-terminal EH (**E**ps15 **H**omology) domain, a central region containing two adjacent proline-rich regions and a carboxy-terminal portion mediating the binding to RalBP1. Via its EH domain, POB1 associates with Eps15 and Epsin [4,5].

The observed interactions with proteins involved in the endocytic pathway and the report that over-expression of the EH domain or the RalBP1 binding region inhibits EGF and insulin internalization, implicates POB1 in receptor endocytosis [4]. Accordingly, POB1 is tyrosine-phosphorylated in response to EGF in COS cells and co-purifies with activated EGF receptor [1]. Furthermore POB1 co-immunoprecipitates with over expressed Grb2 (growth factor receptor-bound protein 2) and with PAG2 (DDEF1), a paxillin-associated protein, at endogenous levels. The sequence responsible for this latter interaction was mapped to a proline-rich motif (_423_PSKPIR_428_) that binds to the SH3 domain of PAG2 [6].

Because of its links to receptor endocytosis and to actin cytoskeleton remodeling, POB1 has been proposed as a molecular scaffold recruiting proteins involved in vesicular trafficking and linking them to actin cytoskeleton.

In addition, more recently, it has been reported that loss of POB1 expression in prostate cancer cells results in deregulation of growth factor signaling, while an increase of POB1 expression has been correlated with silencing of EGF signaling. Moreover, POB1 isoform 2 down-regulation has been observed during progression of prostate cancer from androgen to EGF dependency [7-9].

Although this scattered information suggest a central role of POB1 in the complex regulatory mechanisms modulating growth factor response, no simple model has been put forward to explain all the observations.

In this work, we show that, in addition to the amino-terminal EH domain and the carboxy-terminal RALBP1 binding region, a third central area of POB1, containing motifs recognized by the SH3 domains of Grb2 and Amphiphysin II and by the 14-3-3 family proteins, plays a role in modulating receptor endocytosis. Over-expression of this POB1 fragment negatively affects epidermal growth factor receptor endocytosis by titration and delocalization of essential components. Conversely, point mutations in the conserved binding motifs eliminate the dominant negative effect on receptor endocytosis. These data provide support for a role of POB1 as a regulator of growth factor receptor endocytosis and corroborate its suggested role as a tumor suppressor.

## Results

### Expression of the POB1 proline rich region from Pro308 to Vel365 inhibits EGFR endocytosis

**Figure 1:**
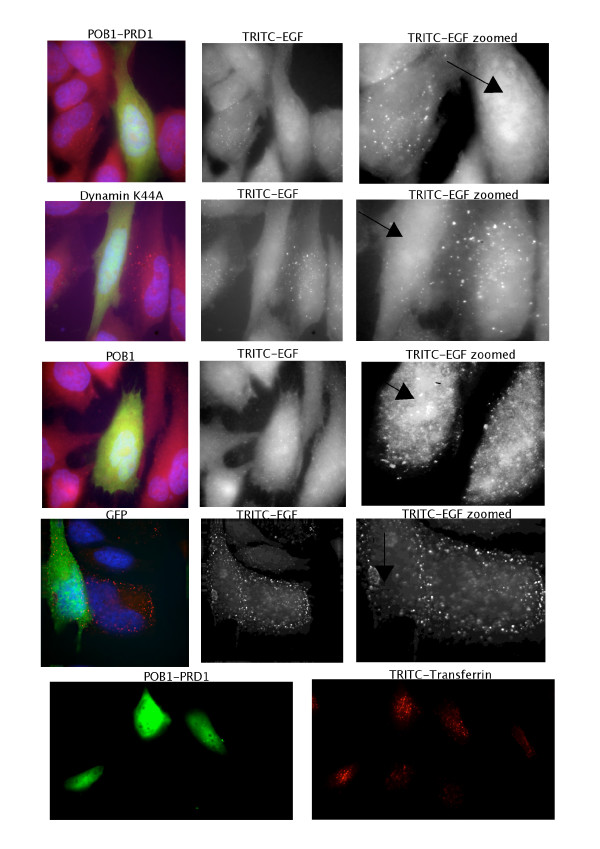
**Expression of the proline rich region of POB1 has a dominant negative effect on EGF endocytosis but it does not affect transferrin endocytosis**. HeLa cells were transiently transfected with GFP-POB1 PRD1 (308–365) or GFP-POB1 full length or, as controls, GFP-Dynamin K44A or GFP alone. Cells were serum starved and incubated with TRITC labeled EGF for 20 minutes at 37°C. Cells were fixed, without acid wash, and observed on an Olympus IX 70 with Nanomover^® ^and softWoRx Delta Vision. Transfected cells are visible in the 528 nm channel as green colored. TRITC-EGF was visualized at 617 nm and is colored in red. Endocytosed vescicles with TRITC-EGF, visible as fluorescent spots, are not detectable in cells over expressing the mutated Dynamin K44A or the central proline rich region of POB1 (POB1 PRD1). A black arrow points the transfected cells in the zoomed sections. HeLa cells transfected with GFP-POB1 PRD1 (308–365) were serum starved and incubated with TRITC-Transferrin (100 mg/ml). POB1-PRD1 transfected cells are visualized as above, internalized transferrin is visible as fluorescent red spots.

While expression of full-length POB1 in A431 cells does not affect either binding or internalization of EGF, over-expression of either the EH domain or the C-terminal region of POB1 affects the ligand dependent internalization pathway of EGF and insulin without interfering with the constitutive transferrin pathway [4]. We were interested in assessing whether the POB1 proline-rich region plays an additional role in the ligand-dependent endocytosis of EGFR. To this end, we transiently transfected HeLa cells with green fluorescent protein (GFP) fusion constructs expressing either the POB1 isoform 2 full length or PRD1, the POB1 proline rich region from Pro308 to Val365 (Fig. 1). As controls we performed parallel transfections with two plasmids expressing GFP or GFP-Dynamin K44A, a dynamin mutant that is an established inhibitor of both EGF and transferrin endocytosis [10]. Transfected cells were serum starved and subsequently incubated with TRITC labeled EGF or transferrin to monitor the two internalization pathways. As already reported, the ectopic expression of full length POB1 had no influence on transferrin or EGF endocytosis [4]. Similarly, POB1 PRD1, the construct expressing a POB1 fragment from amino acid 308 to 365, did not affect the constitutive transferrin endocytic pathway. On the other hand, HeLa cells over-expressing the POB1 PRD1, when treated with TRITC-EGF, have a much reduced number of internalized EGF-stained vesicles and show a diffuse fluorescence, resembling Dynamin K44A expression, indicating that POB1-PRD1 over-expression interferes with EGF endocytosis, (Fig. 1, first row). This phenotype has been observed in five independent experiments with a penetrance of 65% of transfected cells. In similar control experiments, over-expression of Dynamin K44A mutant inhibited EGF internalization with a penetrance of 75%.

### The C-terminal SH3 domain of Grb2 and the SH3 domain of Amphiphysin II bind to full-length POB1 and to POB1 PRD1 (308–365)

To further characterize the interaction network centered on POB1, we sought to isolate proteins binding the proline-rich core region. The POB1 central region from Pro308 to Val365 was expressed in bacteria as a GST fusion protein (GST-POB1 PRD1), immobilized on glutathione-sepharose and used as a bait for affinity selection of a human brain cDNA library displayed on lambda phage [11]. The selected phage clones were sequenced and shown to encode either the Src homology 3 domain (SH3) of Amphiphysin II or the carboxy-terminal SH3 domain of the molecular adaptor Grb2, which was formerly shown to bind tagged POB1 in a GST pull-down assay [1].

**Figure 2:**
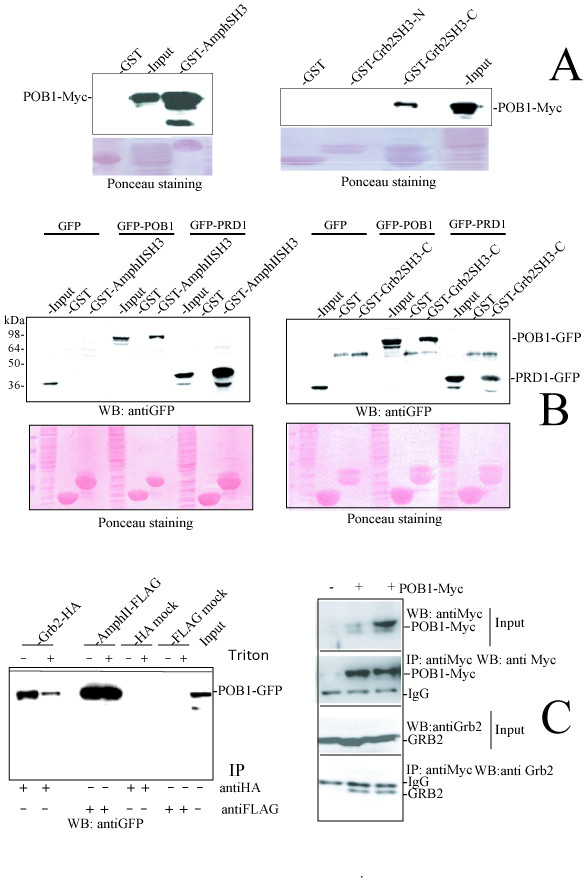
**A) The SH3 domain of Amphiphysin II and the carboxy-terminal SH3 of Grb2 bind full length POB1 in pull down experiments**. Lysates of 293 human embryonic kidney cells (HEK293), transiently transfected with POB1-Myc, were incubated with GST-SH3 of Amphiphysin II (AmphIISH3), GST-SH3 ammino-terminal of Grb2 (Grb2SH3-N), GST-SH3 carboxy-terminal of Grb2 (Grb2SH3-C) and GST alone, adsorbed to glutathione-Sepharose 4B beads. The retained proteins were separated by SDS-PAGE and probed with an anti-Myc antibody (for POB1). The input is 10% of the affinity-selected lysate. The AmphiphysinII SH3 binds 25% of the input while the carboxy-terminal SH3 of Grb2 binds 4%. The ammino-terminal SH3 of Grb2 does not bind POB1. The Ponceau staining shows the GST fusions and the input lysates. **B) The SH3 interacting motif of POB1 is contained in the 308–365 region**. HEK293 cells were transiently transfected with the indicated POB1 constructs expressed as fusions to GFP protein: GFP (lanes 1, 2, 3), GFP-POB1 full length (lanes 4, 5, 6) and GFP-PRD1, the poly-proline rich PRD1 region of POB1 from residue 308 to 365 (7, 8, 9). Cellular extracts were incubated with GST-SH3 of AmphiphysinII (left) or GST-SH3 carboxy-terminal of Grb2 (right) and GST alone. The retained proteins were subjected to SDS-PAGE and probed with an anti-GFP antibody (for POB1 or PRD1). The sample loaded in the input lane is 5% of the material used for affinity selection. A faint unspecific band is visible between the POB1 full length and the PRD1. POB1 PRD1 (308–365) is bound by both the SH3 tested: Amphiphysin II (last lane in leftmost gel) and Grb2 (last lane in rightmost gel). The Ponceau staining shows the GST fusions and the input lysates. **C) POB1 full length co-immunoprecipitates with full length Amphiphysin II and endogenous Grb2**. Left: POB1-GFP was co-expressed in HEK293 cells together with Grb2-HA or Amphiphysin II-FLAG (Amph-FLAG). Cellular extracts were immunoprecipitated (IP) with anti-HA antibody (for Grb2) or anti-FLAG resin (for Amphiphysin II), respectively and the co-immunoprecipitated POB1 was revealed with an anti-GFP antibody. Two lanes for each immuno-precipitation correspond to the same experiment repeated twice but at different washing stringency (without or with 1% Triton). Mock HA and FLAG lanes represent controls transfected with empty vectors. In the input lane we have loaded 1.5% of the cell lysate used for the immunoprecipitation. Right: An empty vector (lane 1) and two independent transfections of POB1-Myc were carried out in HEK293 cells (lanes 2, 3). Upper panel: POB1-Myc in cell lysate was revealed with an anti-Myc antibody (5% of the total cell lysate). Second panel: Cells extracts were immunoprecipitated with anti-Myc (for POB1) and the immunoprecipitated POB1 (an amount corresponding to 20% of the total lysate) were revealed with an anti-Myc antibody; Third panel: the cell lysate was probed with anti-Grb2 antibody, to detect endogenous Grb2 (1% of the total lysate). Panel below: Cells extracts were immunoprecipitated (IP) with anti-Myc (for POB1) and the co-precipitated material was blotted (WB) with an anti-Grb2 antibody to detect endogenous Grb2 co-immunoprecipitated by POB1.

To support these findings, we prepared a cell extract from HEK293 cells transfected with a plasmid encoding Myc-tagged POB1. The extract was incubated with the two SH3 domains of Grb2 (amino- or carboxy-terminal) or the one of Amphiphysin II, expressed as GST fusion proteins. The proteins retained by the affinity resin were separated by SDS-PAGE and probed with an anti-Myc antibody (Fig. 2A). Full length POB1 was only bound by the Grb2 carboxy-terminal SH3 or by the Amphiphysin II SH3 domain, whereas the amino-terminal SH3 domain of Grb2 did not retain the Myc tagged POB1. In a similar experiment the SH3 domains of Grb2 and Amphiphysin II were able to bind to the full length POB1 and to the proline rich domain, PRD1 region 308–365, of POB1, expressed as GFP fusion proteins in HEK293 cells (Fig. 2B).

### POB1 co-immunoprecipitates in vivo with Amphiphysin II and Grb2

In order to assess whether the interactions between POB1 and Amphiphysin II or Grb2 can take place *in vivo*, we transiently co-transfected HEK293 cells with POB1 fused to GFP, together with either Amphiphysin II-FLAG or Grb2-HA (Fig. 2C, left panel). Cell lysates were immunoprecipitated with anti-HA (for Grb2) or anti-FLAG (for Amphiphysin II) and the immunoprecipitates were revealed with an anti-GFP antibody (for POB1). While POB1 co-immunoprecipitates with both Amphiphysin II and Grb2, none of the control lanes shows any detectable POB1 immunoprecipitation.

Moreover, ectopically expressed POB1-Myc was also found to co-immunoprecipitate endogenous Grb2 in HEK293 cells (Fig. 2C right, lower panel). The co-immunoprecipitation of endogenous POB1 full length and endogenous Grb2 is also detected in HeLa cells in an EGF independent manner (Additional file 1).

The interaction of POB1 with Amphiphysin II is consistent with the co-localization of POB1 with clathrin, since Amphiphysin II is a clathrin ligand [12] (Additional file 2). The localization of POB1 in HeLa cells is also not influenced by EGF addition (Additional file 2).

### Arg344 is an important binding determinant in the motifs recognized by the SH3 domains of Amphiphysin II and Grb2

**Figure 3:**
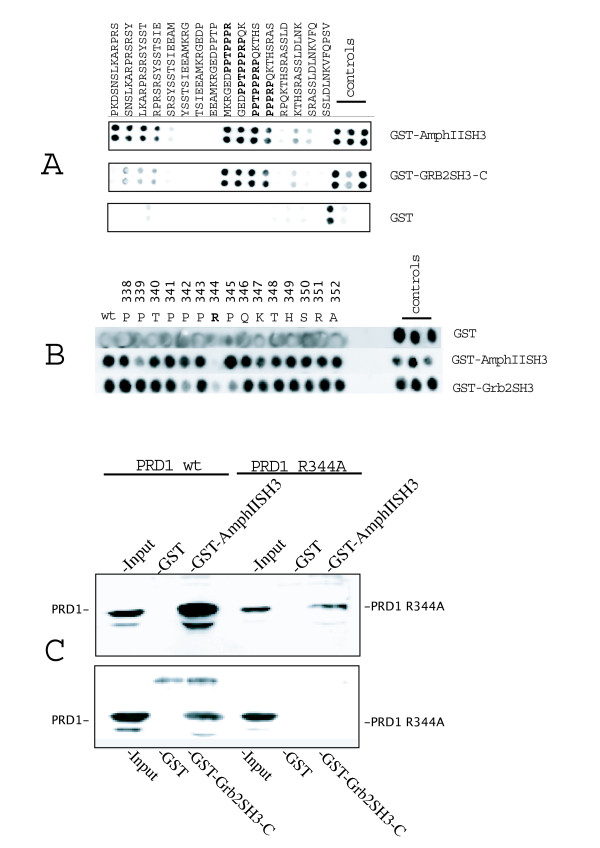
**The SH3 domains of Grb2 and Amphiphysin II bind the POB1 _338_PPTPPPRP_345 _peptide and the mutation R344A squelches the interaction**. **A**). The POB1 PRD1 (P308-V365) region was synthesized as 16 overlapping thirteenmers on a cellulose membrane. The membranes were probed with the SH3 domains of Amphiphysin II (AmphIISH3) or the carboxy-terminal SH3 of Grb2 (Grb2SH3-C) expressed in bacteria as GST fusions. GST alone is used as a negative control. Anti-GST antibodies were utilized to identify bound proteins. The peptide sequences are indicated. The best binding peptides are shown in bold. The three positive control peptides are: GST binding peptides (positive control for GST and secondary antibody) and two peptides containing SH3 interacting motifs. Each binding experiment was carried out in duplicate. The sequence in bold correspond to the best binders. The best peptide 338PPTPPPR344 shows an intensity above 11.000 arbitrary units. Another peptide containing the sequence 214LKARPS219 binds to the SH3 of Amphiphysin, albeit producing a spot intensity around 9000 arbitrary units. Quantitative data are available in additional file 3. **B**) The POB1 fifteenmer peptide (_338_PPTPPPRPQKTHSRA_352_), containing the 338PPTPPPR344 binding motif, was systematically mutagenized by introducing an alanine at each of the indicated 15 positions. The upper membrane was probed with GST as a control, the middle one with the SH3 domain of Amphiphysin II, the bottom one with the carboxy-terminal SH3 of Grb2 fused to GST. The substitution of arginine 344 with alanine reduces the binding of Amphiphysin-SH3 down to 2.5%; a substitution of proline 339 with alanine reduces the binding to 31%. A reduction in Grb2 binding is observed when arginine 344 is substituted with alanine (13%) and when Pro343 or Pro 435 are substituted with Ala (30%). Supplemental quantitative data are provided in additional file 4. **C**) HEK293 cells were transiently transfected with plasmids expressing GFP fused to the proline rich domain PRD1 of POB1 (lane 1, 2, 3) or with an equivalent construct directing the synthesis of the POB1 PRD1 region mutated in Arg344 (PRD1 R344A), in lanes 4, 5, 6. Cell extracts were adsorbed to glutathione resins containing either the SH3 of Amphiphysin II (top gel) or the C-terminal SH3 of Grb2 (bottom gel). Adsorbed proteins were identified with anti GFP antibodies (for POB1 PRD1). Input is 3% of the lysate utilized in the GST pull down.

To identify the recognition motifs bound by the Grb2 and the Amphiphysin II SH3 domains, we first synthesized, on a cellulose membrane, sixteen overlapping peptides (13 amino acid long), spanning the POB1-PRD1 sequence from residue Pro308 to Val365 (Fig. 3A) [13]. The peptides were then probed with the purified SH3 domains of Amphiphysin II and Grb2, fused to GST. This approach established the sequence _338_PPTPPPRP_345 _as the one binding with the highest affinity both SH3 domains (Fig. 3A and additional file 3, for quantitative analysis). Another peptide containing the sequence LKARPS binds to the SH3 of Amphiphysin II, albeit producing a spot intensity 30% lower than the best binder. Next, we systematically mutagenized the pentadecapeptide _338_PPTPPPRPQKTHSRA_352_, containing the best binding motif as established above, by replacing each residue with an alanine (Fig. 3B and additional file 4). This approach identified Arg344 as the main binding determinant for both SH3 domains. Interestingly, aside from this common main determinant, the binding of the Amphiphysin II SH3 domain is more sensitive to substitutions in Pro339 while the binding of the SH3 domain of Grb2 is affected by substitutions in Pro342 and Pro345. This suggests that the two domains bind the same sequence but in a different mode. To validate Arg344's essential role in the interaction between POB1 and the SH3 domains of Amphiphysin II and Grb2, we constructed a variant of the GFP-PRD1, encoding a PRD1 fragment in which Ala replaces Arg344 (Fig. 3C). This POB1 construct was transiently transfected into HEK293 cells and cell lysates were incubated with GST-Amphiphysin II SH3 or GST-Grb2 carboxy-terminal SH3. As shown in Fig. 3C, the Amphiphysin II SH3 domain binds both to GFP-PRD1 and to GFP-PRD1 R344A. However, the recovery of GFP-PRD1 R344A is less efficient, confirming that the mutation Arg344Ala affects this interaction. The Grb2 carboxy-terminal SH3 domain, on the other hand, does not bind GFP-POB1 PRD1 Arg344Ala at all.

These results map the target of the SH3 domain of Amphiphysin and the carboxy-terminal SH3 of Grb2 to a short peptide flanking Arg344 in POB1.

### Endogenous 14-3-3 isoforms bind to the POB1 PRD1 region in a phosphorylation dependent manner

**Figure 4:**
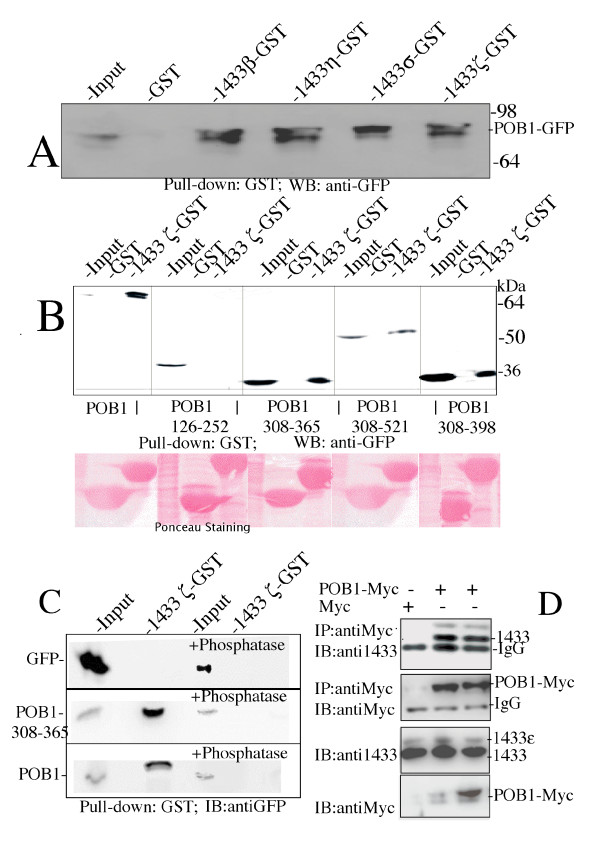
**Different 14-3-3 isoforms bind to the PRD1 region of POB1 in a phospho-dependent manner**. **A) **Lysates of HEK293 cells, transiently transfected with POB1 fused to GFP, were incubated with four different isoforms of human 14-3-3 fused to GST adsorbed to glutathione-sepharose 4B beads. The retained proteins were separated by SDS-PAGE and probed with anti-GFP antibody (for POB1). 5% of the extract used in the pull down was loaded in the input lane. **B) **HEK293 cells were transiently transfected with different POB1 deletion constructs fused to GFP: POB1 full length (1–521 amino acids), POB1 EH domain (126–252), POB1 proline rich region 1 PRD1 (308–365), POB1 pro-rich and carboxy-terminal (308–521) and POB1 proline-rich region (308–398). Cell lysates were incubated with GST-14-3-3 ζ and GST alone, adsorbed to sepharose resin. The retained proteins were separated by 10% SDS-PAGE and probed with anti-GFP antibodies (for POB1). 5% of the extract adsorbed to the affinity resins was analyzed in the input lane. Figures in the deletion constructs refer to the amino acid residues of the POB1, short isoform, Swiss Prot entry Q8NFH8-2. The Ponceau staining shows the GST fusions and the input lysates. **C)**. HEK293 cells were transiently transfected with vectors expressing GFP protein alone (panel above), POB1 PRD1 308–365 (middle panel) or full length POB1 (below) fused to GFP and treated as in B. 50% of each cell lysate was incubated with the Ser/Thr λ-phosphatase for 2 hours at 30°C before incubation with GST-14-3-3 ζ. Input lysates are 5% of the lysates adsorbed to the affinity resin. POB1 PRD1 and the full length POB1 are revealed by anti-GFP antibody. Upper panel is a negative control of mock vector expressing only GFP. **D) **A plasmid expressing Myc tagged POB1 was used to transfect HEK293 cells in two independent replica experiments. Cells extracts expressing the empty plasmid (lane 1) or the Myc tagged POB1 (lanes 2 and 3) were immunoprecipitated with anti-Myc antibodies (for POB1). The proteins immunoprecipitated by anti-Myc (20% of the total lysate) were separated by SDS PAGE and probed with anti-14-3-3 (upper panel) or anti-Myc antibodies (second panel). In the upper panel, the endogenous 14-3-3 proteins co-immunoprecipitating with POB1 are revealed by anti-14-3-3. Also the slower migrating band of the longest 14-3-3 isoform, epsilon, is efficiently recovered. The control of the immunoprecipitated POB1 is shown in the second panel. The endogenous 14-3-3 proteins in the input lysate are revealed by anti-14-3-3 in the third panel where the slower migrating band of the longest 14-3-3 isoform epsilon is also visible (5% of total lysate). The ectopically expressed POB1-Myc is revealed with anti-Myc antibodies, in the bottom panel (1% of total lysate).

Upon sequence examination by ELM (Eukaryotic Linear Motif) search, POB1 contains three putative 14-3-3 binding sites centered on Ser121, Ser322 and Ser354 . In order to validate this prediction, we performed pull-down experiments using, as baits, four members of the human 14-3-3 family fused to GST. All the 14-3-3 isoforms that we have tested (β, η, σ, ζ) were able to bind with comparable affinities to a POB1-GFP full-length chimera over-expressed in HEK293 cells (Fig. 4A). It should be noted that 14-3-3 proteins seem to enrich for a slower migrating band, probably a phosphorylated form of POB1.

In order to better define the POB1 region involved in the interaction, we engineered a series of plasmids directing the synthesis of deletion mutants of the POB1 protein: POB1 126–252 contains the EH domain, POB1 308–365 encompasses the first poly-proline region, PRD1, POB1 308–398 comprises two adjacent proline rich regions while POB1 308–521 further extends to the carboxy-terminal region including the RalBP1 binding domain (Figure 7). The pull down experiment in Figure 4B confirms the interaction of 14-3-3 with the carboxy-terminal portion of POB1 (from 308 to 521) and restricts the minimal essential binding site to the PRD1 region of POB1 (residues 308 to 365) including both Ser322 and Ser354.

In order to assess whether POB1 binds to endogenous 14-3-3, we transiently transfected HEK293 cells with Myc-POB1 in two independent experimental repeats (Fig. 4D). Cell lysates were immunoprecipitated with anti-Myc antibodies (for POB1) while the co-immunoprecipitated endogenous 14-3-3 proteins were detected with specific antibodies. The results in Figure 4D, upper panel, show that endogenous 14-3-3 proteins, including the largest isoform (epsilon), co-immunoprecipitate with POB1.

Since most 14-3-3 interactions are phosphorylation dependent [14-17]we tested whether the interaction between 14-3-3 and POB1 PRD1 is mediated by a phospho-peptide. We performed a pull down experiment as described in Fig. 4B after incubating half of the cell extract with λ-phosphatase, active towards phosphorylated serine and threonine (Fig. 4C). When treated with phosphatase, neither the full length POB1 (bottom panel, lane 4) nor the POB1 PRD1 region (middle panel, lane 4) is efficiently recovered by 14-3-3. The same absence of co-immunoprecipitation occurred after wortmannin treatment suggesting an involvement of Akt Ser/Thr kinase (data not shown).

Taken together, these results suggest that the interaction between POB1 and the 14-3-3 proteins can occur *in vivo*, the recognized motif is encompassed in the region 308–365 of POB1 and the binding is Ser/Thr phosphorylation dependent.

Coimmunoprecipitation of POB1 with the isoform ζ is observed also at endogenous levels in HeLa cells (Additional file 1).

### Characterization of the 14-3-3 binding site: Ser354

**Figure 5:**
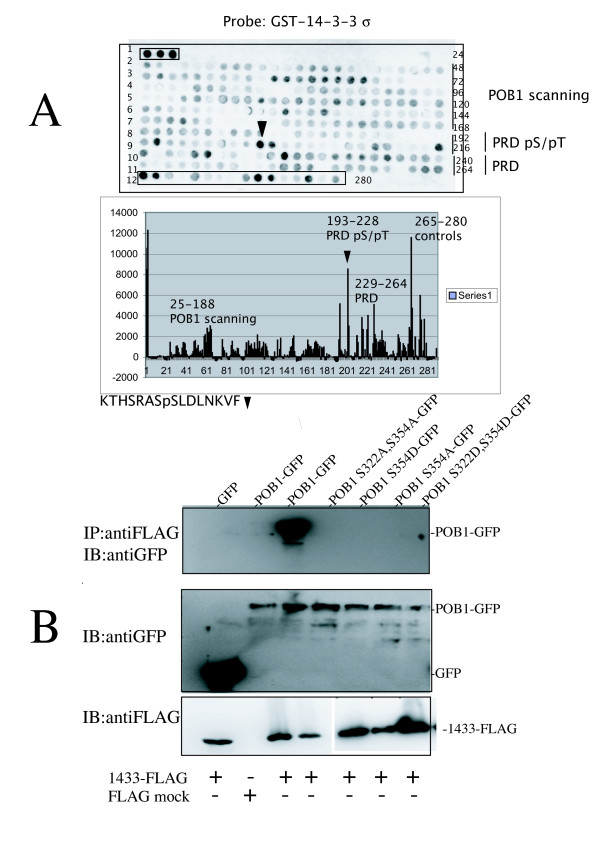
**14-3-3 proteins bind a phospho-peptide centered on Ser354**. **A) **The sequence of POB1 was synthesized by the SPOT method in an array of 15 residue long peptides on a cellulose membrane. Numbers 1 to12 on the left of the membrane are the rows. Numbers on the right (24 to 280) are the numbers of the spotted peptides, reported in the intensity diagram. The first three intense spots in row 1 contain peptides RRFRsLPAAH, RRHRsAPGVR and RRSRsFPVTF (where 's' represents a phosphorylated serine) that serve as positive controls of 14-3-3 interactions. The peptides No. 25-188 (row 2–8) are 15 mers whose overlapping sequences span the POB1 sequence from Leu181 to the carboxy-terminal Leu521. Rows 9–11 contain 15 mer-peptides whose sequence is centered on each single serine or threonine in the POB1 PRD1 (308–365). The peptides from No.193 to No. 228, in row 9 and the first 12 peptides in row 10, contain a centered phospho-serine or phospho-threonine. Their unphosphorylated counterparts, No. 229 to No. 264, occupy the last twelve spots in rows 10 and the 24 spots in row 11. Row 12 contains positive and negative control peptides. The membrane was incubated with purified GST-14-3-3 sigma and probed with anti-GST antibodies. The control membrane, incubated with GST, was blank. The intensity of the interaction was revealed with a peroxidase coupled secondary antibody. The POB1 peptide showing the strongest signal (black triangle) is peptide number 203, whose sequence 347KTHSRASsLDLNKVF361, is centered on phosphorylated Ser354 and shows a signal intensity of 8504 arbitrary units (a.u.), comparable with the strongest positive controls. Its non-phosphorylated counterpart is spot number 238 (intensity 991 a.u.). The peptide centered around Ser322 is No.196 (when phosphorylated) and shows a signal of 491 a.u., while its unmodified counterpart is No.232, 1400 a.u. A comparable result was obtained by probing the same array with GST-14-3-3 isoform zeta. The file reporting the quantitative analysis is in additional file 5. The graph is shown reporting the peptide number referred to the position on the membrane and the intensity measured. **B) **3xFLAG 14-3-3 zeta was co-expressed in HEK293 with GFP or different constructs expressing POB1 full length-GFP wild type or bearing the indicated mutations of Ser322 or/and Ser354 into alanine (A) or aspartate (D). Lane 1 and 2 are negative controls: in lane 1, the GFP empty vector was co-transfected with 14-3-3; in lane 2, POB1-GFP is co-transfected with the 3xFLAG empty vector. Upper blot: cell lysates were immunoprecipitated with anti-FLAG antibodies (for 14-3-3) and the co-immunoprecipitated POB1-GFP was revealed with anti-GFP antibodies. POB1-GFP is recovered only when Ser354 is not mutated. In the two gels below, 5% of the cell extract utilized in the immunoprecipitation was analyzed by gel electrophoresis and probed with anti-GFP (for POB1) antibodies or anti-FLAG (for 14-3-3) to assess POB1, GFP and 14-3-3 in input.

As a means to map the 14-3-3 binding site, we have first fragmented the POB1 PRD1 region (from residue 308 to 365) into fifteen amino acid long overlapping peptides synthesized on a cellulose membrane (Fig. 5A, rows 2–8, peptides 25–188). Since 14-3-3 proteins preferentially bind motifs containing phosphorylated serines or threonines, we have synthesized in parallel all of the PRD1 region's 15 mers containing a central serine or a threonine (i.e., 36 peptides, from 193 to 228). These peptides were synthesized both in the phosphorylated (row 9-and half of row 10; labeled PRD pS/pT) and in the unphosphorylated forms (half row 10- row 11; peptides 229–264, labeled PRD). Fig. 5A shows the results obtained by probing the corresponding peptide array, with the human 14-3-3 sigma isoform (Intensity diagram in Fig 5A and additional file 5 for quantitative data). Among the POB1 peptides the one showing the strongest signal (indicated by a black triangle, peptide No.202) is _347_KTHSRASpSLDLNKVF_361 _(where pS represents phosphoserine 354), although its sequence does not match a typical class 1 or class 2 14-3-3 consensus [14,15]. Similar results were obtained by probing comparable membranes with the zeta isoform (data not shown). This result identifies the phospho-Ser354 as an important determinant in the 14-3-3 binding site and confirms the requirement for a phosphorylated serine since the same peptide, in the un-phosphorylated form, does not bind 14-3-3 (peptide No. 238). The second predicted 14-3-3 binding site, flanking Ser322, binds 14-3-3 much less tightly when compared with Ser354.

To confirm the functional relevance of the 14-3-3 binding site characterized *in vitro*, we transiently co-transfected HEK293 cells with 3xFLAG-14-3-3 isoform zeta and POB1-GFP, bearing mutations in Ser354 and in Ser322. The two serines are within two sequences resembling 14-3-3 type 2 motifs (RSRS_322_YS and RASS_354_LD) and are predicted 14-3-3 ligands by the Motif Scan software at scansite.mit.edu. However, since the phospho-peptide centered on Ser322 did not bind 14-3-3 in the membrane-binding assay, we focused on the Ser354 motif by constructing Ser354Ala and Ser354Asp mutants. In addition, we also constructed double mutants by mutating Ser 322 (Fig. 5B). Cell lysates were immunoprecipitated with anti-FLAG antibodies (for 14-3-3) and the co-immunoprecipitated POB1 was revealed with anti-GFP antibodies. As shown in the upper panel (Fig. 5B), wild type POB1 co-immunoprecipitates with 14-3-3 (lane 3), while, substituting Ser354 with Ala (lane 6) is sufficient to abolish binding. Replacing Ser354 with a negatively charged residue, aspartate, cannot mimic the 14-3-3 binding motif (lane 5). These results prove that phosphorylation of Ser354 in POB1 dictates 14-3-3 binding. The substitution of Ser354 with aspartate does not recapitulate binding, as often seen in 14-3-3 interactions with phosphorylated substrates, as, for example, RGS or keratin [18,19]. These data show that mutation of Ser354 in POB1 abolishes interaction with 14-3-3.

### Mutations affecting POB1 PRD1 binding to SH3 or 14-3-3 suppress the dominant negative phenotype

To correlate binding to function, we decided to ask if the expression of POB1 PRD1 mutated in the SH3 and the 14-3-3 binding motifs is able to affect EGF induced receptor endocytosis.

**Figure 6:**
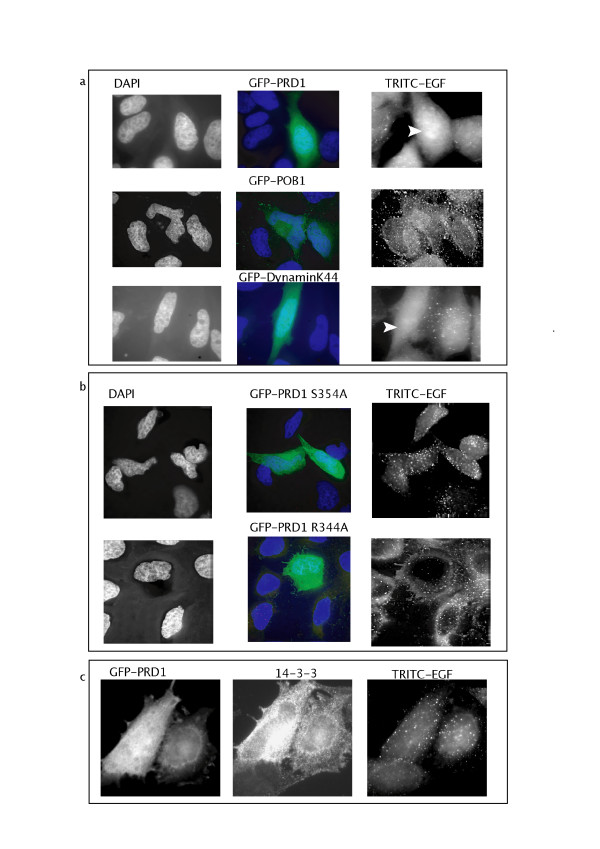
**Mutations in Ser354 or Arg344 abolish the dominant negative effect of POB1-PRD1 on EGF endocytosis**. **a) **HeLa cells were transiently transfected with GFP-POB1 PRD1, GFP-POB1 or GFP-Dynamin K44, serum starved and incubated with TRITC-EGF for 20 minutes at 37°C. Cells were fixed and observed on an Olympus IX 70 with Nanomover^® ^and softWoRx DeltaVision. GFP transfected cells are visible in the 528 nm channel (green). TRITC EGF was visualized at 617 nm (fluorescent spots). Nuclei were visualized by DAPI staining at 457 nm (blue). Transfected cells that do not show endocytic vescicles are indicated by a white arrow. **b) **HeLa cells were transiently transfected with GFP-POB1 PRD1 S354A or GFP-POB1 PRD1 R344A and treated as above. Transfected cells are visible in the 528 nm channel as green cells. These cells show a wild type distribution of fluorescent endocytic vesicles (TRITC-EGF visualized at 617 nm). Nuclei were visualized by DAPI staining at 457 nm (blue). **c)** Overexpression of 14-3-3 abolish the dominant negative effect of POB1-PRD1 on EGF endocytosis. HeLa cells were transiently transfected with GFP-POB1 PRD1 together with 3xFLAG-14-3-3 ζ. Cells were treated as above. Cells were then permeabilized and treated with Cy5-conjugated antibodies targeting the FLAG epitope of the 14-3-3 proteins, visible at 685 nm. Cells were observed as described above. These cells show a wild type distribution of fluorescent endocytic vesicles (TRITC-EGF visualized at 617 nm).

In contrast with its wild type counterpart (Fig. 6A), the over-expression of GFP-POB1 PRD1 S354A, mutated in the serine residue that dictates 14-3-3 binding, does not affect endocytosis. (Fig. 6B). Also, cells over expressing POB1 PRD1 R344A, which is defective in binding to Amphiphysin II and Grb2 SH3, do not show any interference in the wild type EGF internalization (Fig. 6B). These results demonstrate that the POB1 proline-rich region acts as a dominant negative for EGF internalization and that this negative effect can be rescued by mutations in the residues that affect binding to 14-3-3 or to specific SH3.

Interestingly, the normal phenotype of endocytosis is restored upon co-transfection of the wild type POB1 PRD1 region with 3xFLAG-14-3-3 ζ in over-expression (Fig. 6C). We conclude that over-expression of 14-3-3 ζ removes the endocytosis block caused by the dominant negative effect of the PRD1 region of POB1.

## Discussion

The observation that over-expression of the EH domain or of the carboxy-terminal RalBP1 binding region of POB1/REPS2 results in a 30–40% reduction of EGF or insulin internalization has linked POB1 to the molecular machinery modulating regulated endocytosis [4]. More recently, it was reported that loss of POB1 expression during human prostate cancer progression, from androgen-dependent to growth factors dependent, results in loss of control of cell growth signaling while induced expression of POB1 causes a reduction of several EGF-responsive genes (e.g., Fos and Jun). In accordance, we find that an increase of POB1 isoform 2 expression correlates with a decrease of EGF-induced phosphorylation of Erk1–2 and Shc (Additional file 6). Moreover, POB1 isoform 2 down-regulation was observed during the progression of prostate cancer [7-9].

In our study we confirm the involvement of POB1 in EGF receptor endocytosis and we provide evidence for a functional role of the central proline-rich region. In fact, we observed that over-expression of the proline-rich region of POB1 (308–365) interferes with EGFR endocytosis while leaving transferrin uptake unaffected (Fig. 1). This suggests that, in addition to the N and C-terminal regions, the central proline-rich domain (PRD1) of POB1, from Pro308 to Val365, also plays an important function in regulated endocytosis and that over-expression of this portion of POB1 disturbs the dynamics of protein interactions by titration of elements required for regulated endocytosis.

By panning phage displayed cDNA libraries, with a POB1 construct containing the proline-rich domain PRD1, we enriched for phages displaying the carboxy-terminal SH3 domain of Grb2 and the SH3 domain of Amphiphysin II. These in vitro identified physical interactions were further confirmed in vivo, with full length POB1 (Fig. 2). A complex between POB1 and Grb2 has already been reported [6], We add that POB1 can bind Grb2 at endogenous levels (Fig. 2 and Additional file 1) and we report that the interaction occurs by means of the carboxy-terminal SH3 domain of Grb2. Interestingly the Grb2 binding motif in POB1 (_341_P**P**P**RP**_345_) is exactly conserved in the Gab motif (_337_P**P**P**RP**P_342_) that is also recognized by Grb2. This motif is also conserved in the highly homologous human protein REPS1. POB1 shows a punctate cytoplasmic localization that is not much affected by EGF induction (Additional file 2). A substantial fraction of POB1 co-stains with the late endosome markers CD63 or LAMP-1, as also found for tyrosine-phosphorylated EGFR, Shc, Cbl and Grb2 [20].

We describe Amphiphysin II as a new POB1 interactor. POB1 cellular colocalization with clathrin is consistent with this interaction, since Amphiphysin II is also a clathrin ligand (Additional file 2) (20). By alanine scanning mutagenesis we have shown that both Grb2 and Amphiphysin II SH3 bind to the same POB1 peptide, however, different residues seem to be the main determinants of recognition specificity. Amphiphysin II binding is affected by changes in Arg344 and Pro339 while Grb2 binding is sensitive to changes in Pro342 and Pro 345 (Fig. 3).

We have characterized a second linear motif of POB1 containing a phosphorylated serine and mediating binding to the 14-3-3 isoforms (Fig. 4). This binding site was mapped approximately ten amino acids away from the SH3 binding motif (Fig. 5). 14-3-3 binding was shown to be sensitive to phosphatase treatment (Fig. 4) and binding was abolished when Ser354 was mutated to Ala or to Asp (Fig. 5). This suggests that POB1 binds 14-3-3 in a phosphorylated form. Significantly, the characterized POB1 peptide sequence HSRASsLDLNK, only displays poor similarity to the classical 14-3-3 binding motifs [15]. In fact, while presenting the conserved Arg at position -3 from the phosphorylated serine, it displays a few uncommon features, such as an Asp at position +2, in place of the preferred Pro. This divergence is not anomalous, given that physiological substrates often bind 14-3-3 through peptide ligands which do not exactly match the most common motifs [21]. Interestingly binding is abolished if Ser353, immediately preceding the phosphorylated Ser354, is also phosphorylated (data not shown). 14-3-3 have been described as forming complexes with more than 200 partners in high throughput studies and potentially engaging 0.6% of the human proteome [22]. Some of these complexes have been implicated in regulating protein membrane trafficking. For instance, 14-3-3 were shown to reduce endoplasmic reticulum (ER) localization, thereby promoting surface expression of membrane proteins [17,23].

It has to be emphasized that 14-3-3 zeta has already been reported as associating with the epidermal growth factor receptor cytoplasmic tail and co-localizing along the plasma membrane with EGFR upon EGF stimulation [24]. Thus a 14-3-3 dimer could bridge POB1 to the EGFR upon EGF induction. Similarly, 14-3-3 proteins appear to be the essential molecular bridge between the alfa1-subunit of the Na+, K+, -ATPase in the plasma membrane and the PI 3-kinase, thus providing the signal to initiate endocytosis of the protein in response to natriuretic hormones [25]. Finally 14-3-3 sigma is phosphorylated in prostate cells in response to EGF [26]. It is interesting to note that the 14-3-3 binding motif HSRxSSLD flanking Ser354 of POB1, that we find essential for interaction with 14-3-3, is conserved in the mouse orthologs and flanks the Ser510 of human REPS1, that is found phosphorylated *in vivo *in A431 cells (Stover DR et al. Phosphosite: ). This serine site is predicted to be phosphorylated by Akt or calcium/calmodulin-dependent protein kinase II gamma, CaMK2G. In agreement, we observed absence of 14-3-3-coimmunoprecipitated POB1 when cells where treated with phosphatase or wortmannin (not shown), an inhibitor of PI3K and consequently of Akt.

To further test the model we assayed the POB1 proline-rich construct harboring the point mutation R344A, which prevents binding of Grb2 and Amphiphysin II and, as expected, we did not observe the dominant negative effect on EGFR internalization (Fig. 6). Thus, the Arg344Ala mutation both affects the formation of the POB1/Grb2 and POB1/Amphiphysin II complexes and also abolishes the dominant negative phenotype affecting endocytosis. This suggests that titration of either SH3 containing partner affects EGFR internalization. Similarly, a POB1 PRD1 construct whose ability to bind 14-3-3 was impaired by a Ser354Ala mutation, was unable to inhibit endocytosis in over-expression conditions (Fig. 6). In addition, we have found that co-expression of 14-3-3 relieves the dominant negative effect of POB1-PRD1 on EGFR internalization (Fig. 6). Mechanistically, 14-3-3 ζ could offset the negative POB1-PRD1 over-expression effect, by binding POB1-PRD1 and by preventing the recruitment and trapping of a second essential physiological ligand.

## Conclusion

**Figure 7:**
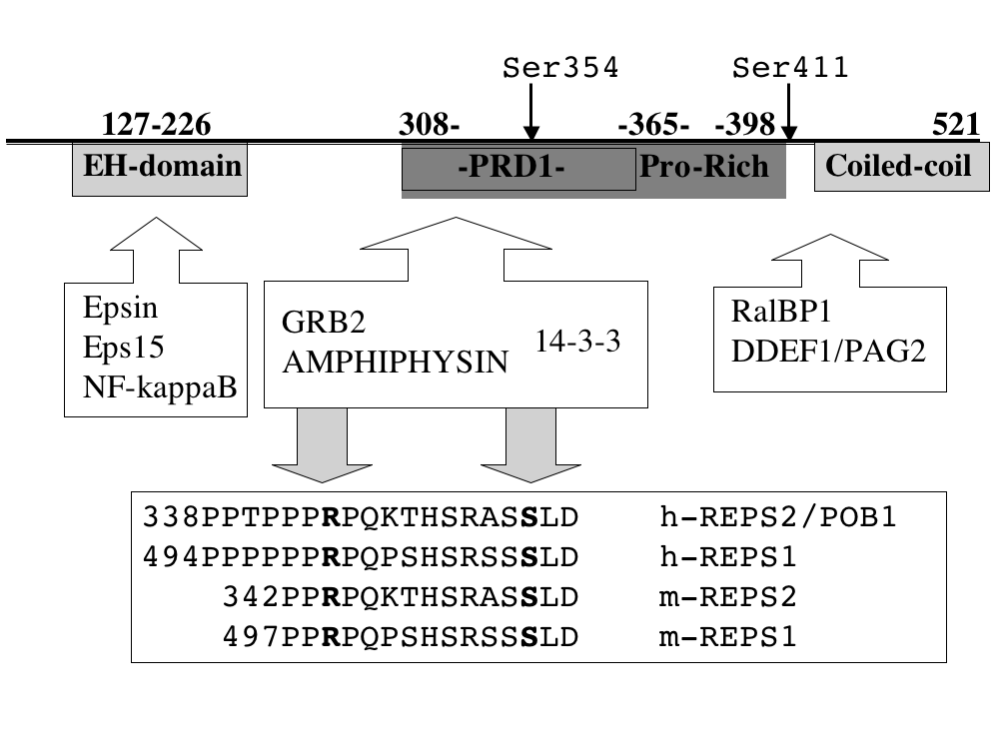
**Outline of POB1/REPS2 and synopsis of its protein interactions**. POB1/REPS2 isoform 2 is represented as a line of 521 residues. The EH domain is from 127 to 226; the central proline-rich region is 308–398; the carboxy-terminal part is a coiled-coil. Evidences presented in this work support interactions involving the first proline rich domain 308–365 (PRD1). The SH3 of Amphiphysin II and the carboxy-terminal SH3 of Grb2 specifically bind a peptide centered around arginine 344, while the 14-3-3 family proteins bind the serine 354, if phosphorylated. The proteins recognized by the EH domain of POB1 are: Epsin [5], Nf-kB [9] and Eps15 [28,4]. The proteins interacting with the distal proline rich domain and the C-terminal domain are RalBP1 (1), and DDEF1/PAG2/ASAP1 whose SH3 domains bind a peptide including P423 and P425 [6]. The phosphorylation of Ser411 by p34 (cdc2) in mitotic phase [29] is reported, the phosphorylation of Ser354 is discussed in this work. The box below shows the consensus sequence for 14-3-3 and SH3 binding in the POB1 human paralogs and mouse orthologs: human REPS1 Q96D71, mouse REPS1 O54916 and mouse REPS2 Q8OXA6. The POB1 sequence coordinates refer to human REPS2 Q8NFH8-2.

We confirm that POB1 plays an active role in controlling the dynamics of ligand-dependent receptor internalization and signaling. This is mediated by several interactions with diverse protein partners [4,5]. In this work, we provide evidence of the central role played by the proline rich region of POB1 through binding to 14-3-3 and to the SH3 of Grb2 and Amphyphisin II (Fig. 7). We observe that these interactions are not EGF dependent and are probably exclusive, since the binding motifs are only nine amino acid apart.

The artificial over-expression of this portion of POB1 affects endocytosis by titration of essential components, leading to formation of partial complexes that are not functional [7]. Thus POB1 acts as a sort of scaffold to enucleate a complex that is required for receptor internalization. The assembly and composition of this complex is finely regulated by protein concentration. Besides contributing to expanding and further characterizing the functional endocytosis "interactome", these results add to the molecular interpretation of POB1 as a tumor suppressor, due to its acting as a scaffold to bridge proteins which are established promoters of endocytosis and, subsequently, of receptor down regulation.

## Methods

### Cell culture and transient transfection

HeLa and human embryonic kidney cell line HEK293 Phoenix were grown at 37°C in a 5% CO_2 _incubator, in DMEM (Gibco/Invitrogen) supplemented with 10% fetal bovine serum (SIGMA-Aldrich), penicillin and streptomycin (Gibco). HeLa cells were transfected with Lipofectamine 2000 reagent (Invitrogen) following manufacturer's instructions while HEK293 cells were transfected with the calcium-phosphate method.

### Plasmids

The plasmid vectors encoding for GFP-POB1, GFP-Dynamin K44A and FLAG-Amphiphysin II were a generous gift from A. Kikuchi, G. Cestra and H. Daub, respectively. The remaining plasmids were constructed by standard recombinant DNA techniques. Briefly, the cDNA encoding for the Amphiphysin II Src homology 3 domain (SH3) was cloned using *Sal*I and *Bgl*II pYEX (modified pGEX-2TK, Amersham) unique sites, and GST-Grb2 carboxy-terminal SH3 was generated by cloning the corresponding cDNA in pYEX *Bam*HI and *Not*I sites. The construct expressing GST-POB PRD1 was generated by amplifying the corresponding POB1 cDNA region and cloning it into *Bam*HI and *Eco*RI pYEX unique sites. The same POB1 cDNA region (POB1/REPS2 Swiss-prot: Q8NFH8, isoform 2, 521 amino acids) was cloned into the *Bgl*II and *Eco*RI sites of pEGFP-C1 vector (BD Bioscience Clontech) for expression in eukaryotic cells. PRD1 region was amplified by PCR with the primers 5'-ATCCAGGATCCCCCAAGGATTCCAACAGT-3' and 5'-TGGTGAATTCCACACTGGGCTGGAAGAC-3' and sub cloned into *Bgl*II and *Eco*RI sites; the PRD1–PRD2 (308–398) fragment was amplified by PCR with the oligos 5'ATCCAGGATCCCCCAAGGATTCCAACAGT-3' and 5'GGCAAGCTTTTACACTTGTTCAGACTGTGA-3' and cloned into the *BglI*I-*Hind*III sites; the NH_2 _terminal-EH region was amplified by PCR with the oligos 5'TGCCTGGATCCATGATGTCAAAGAAT-3' and 5'GATGAATTCGCTGCAGAGTTGGAGGGAGGC and subcloned into the *BglI*I-*Eco*RI sites; the PRO-RICH and carboxyterminal region (308–521) was amplified by PCR with oligos: 5'ATCCAGGATCCCCCAAGGATTCCAACAGT-3' and 5'TGTCTAGACAACACAGTGACCGGACG-3' and cloned into the *BglI*I-*Sal*I sites. Site-directed mutagenesis of GFP-POB PRD1 to obtain GFP-POB1 PRD1 R344A was carried out by assembly of two overlapping DNA fragments obtained by PCR amplification with pairs of complementary primers (R1299 5'ACCCCGCCACCTGCACCACAGAAAACC-3', R1300 5'GGTTTTCTGTGGTGCAGGTGGCGGGGT-3'), each carrying the mutated sequence, and two oligos priming from the 5'- and 3'-ends of the wild type POB1 proline-rich sequence. This latter pair of primers contained the *Bam*HI and *Eco*RI sites for cloning into the pEGFP-C1 vector. The cDNA encoding for POB1 was cloned into pcDNA3.1/Myc-His *Eco*RI and *Not*I unique sites. Finally, HA-Grb2 was constructed by amplifying the cDNA coding for Grb2 and cloning it into *Bgl*II and *Eco*RI sites of pcDNA-HA (Natoli et al., 1997).

The plasmid vectors pFLAG CMV2 encoding the 14-3-3 isoforms β,ε,η,θ,σ,ζ were a generous gift from Takashi Tsuruo. The same isoforms were subcloned in a pCDNA/FRT/TO Invitrogen modified by insertion of a box encoding a 3xFLAG tag and a cloning site suitable for the LIC cloning method (52), flanked by *Hind*III-*BamHI *sites. The 3xFLAG box was inserted utilizing the oligos: 5'CCCAAGCTTACCATGGACTACAAAGACCAT-3' and 5'CGGGATCCCCGTTATCCACTTTCCCCGGGGGATTGGAAGTACAGCTTGTCATCGTCATC-3'. The 14-3-3 isoforms were amplified by PCR and cloned in this box using the oligos:

5'TACTTCCAATCCCCCGGCATGACAATGGATAAAAGT and

5'TTATCCACTTTCCCCGTTAGTTCTCTCCCTCCCC for β;

5'TACTTCCAATCCCCCGGCATGGATAAAAATGAGCTGGTT and

5'TTATCCACTTTCCCCGTTAATTTTCCCCTCCTTCTC for ζ;

5'TACTTCCAATCCCCCGGCATGGATGATCGAGAGGATCT and

5'TTATCCACTTTCCCCGTCACTGATTTTCGTCTTCCA for ε;

5'TACTTCCAATCCCCCGGCATGGGGGACCGGGAGCAGC and

5'TTATCCACTTTCCCCGTCAGTTGCCTTCTCCTGCTT for η;

5'TACTTCCAATCCCCCGGCATGGAGAGAGCCAGTCTGATC and

5'TTATCCACTTTCCCCGTCAGCTCTGGGGCTCCTGGG for σ. The cDNA encoding for GST 14-3-3 β,ε,η,θ,σ,ζ was cloned into the LIC site of a pGEX-2T-K modified by insertion of a box suitable for LIC cloning method in *BamH*I and *EcoR*I sites, by the annealed oligos

5'GATCCCTGTACTTCCAATCCCCCGGGGAAAGTGGATAACGGG-3' and

5'AATTCCCGTTATCCACTTTCCCCGGGGGATTGGAAGTACAGG.

Site directed mutagenesis of GFP-POB1 and GFP-POB1-PRD1 to obtain GFP-POB1 S354A, GFP-POB1 S322A, GFP-POB1 S322/354A and GFP-POB1 PRD1 S354A was carried out by using the QuikChange Site-directed Mutagenesis Kit (Stratagene) with primers R1528 for S354D: 5'ACCCATTCCAGAGCCTCCG**A**CTTGGATCTGAATAAA; R1529 for S322A:

5'GCAAGACCAAGATCCAGAG**C**TTACTCTAGCACC; R1530 for S322D:

5'GCAAGACCAAGATCCAGAG**A**TTACTCTAGCACC; R1532 for S354A:

5'ACCCATTCCAGAGCCTCCG**C**CTTGGATCTGAATAAA. It should be noted that all the residue numbering of POB1/REPS2 refers to the isoform 2, Q8NFH8-2.

### Antibodies

The antibodies used in these experiments were a mouse monoclonal anti-T7 (Novagen), a rabbit polyclonal anti-lambda serum, a mouse monoclonal anti-HA (SIGMA-Aldrich), a mouse monoclonal anti-FLAG (SIGMA-Aldrich), a rabbit polyclonal anti-GFP (Santa Cruz), a mouse monoclonal anti-Grb2, a mouse monoclonal anti-CHC, a mouse monoclonal anti-EEA1, a mouse monoclonal anti-CD63, a mouse monoclonal anti-Lamp2, a mouse monoclonal anti-GM130 (all from Becton Dickinson Transduction Laboratories); rabbit anti 14-3-3 zeta (Santa Cruz); a mouse monoclonal anti 14-3-3 sigma (Santa Cruz); mouse monoclonal anti 14-3-3 (Neomarker); polyclonal anti-REPS2 raised in rabbit (Atlas Antibody). A rabbit polyclonal anti-POB1 serum was generated against a N-terminal part of POB1, tested on western blot in parallel with commercial anti-REPS2. Secondary antibodies used in this work were an alkaline phosphatase-conjugated anti-rabbit IgG, an alkaline phosphatase-conjugated anti-mouse IgG (both from SIGMA-Aldrich), a peroxidase-conjugated anti-mouse and anti-rabbit IgG, a Rhodamine Red-X-coupled anti-rabbit (all from Jackson ImmunoResearch). Cy5-coupled anti mouse (from Jackson ImmunoResearch) and an Alexa Fluor 488-coupled anti-mouse (Molecular Probes).

### Pull-down experiments

GST and GST-fusion proteins (100 μg) expressed in bacteria and adsorbed to glutathione-sepharose 4B beads (Amersham) were incubated for two hours at 4°C with 1 mg of HEK293 cellular extract in JS lysis buffer (50 mM Hepes, 150 mM NaCl, 1% Glycerol, 1% Triton-X100, 1.5 mM MgCl_2_, 5 mM EGTA, 1 mM PMSF, 10 μg/ml Leupeptin, 10 μg/ml Aprotinin, 1 μM NaVO_4_). Beads were washed three times in ice-cold PBS. Bound proteins were electrophoresed on a 10% polyacrylamide gel, transferred onto nitrocellulose membranes and detected with specific antibodies.

### Protein dephosphorylation

To release phosphate groups from serine or threonines, cell lysis was performed by adding JS buffer without phosphatase inhibitor (50 mM Hepes, 150 mM NaCl, 1% Glycerol, 1% Triton X-100, 1.5 mM MgCl2, 5 mM EGTA, protease inhibitor cocktail Sigma-Aldrich 200×). The cell extracts were incubated with 300 units of phosphoSer/Thr λ protein phosphatase (for 500 micrograms of total protein extract) for 2 h at 30°C. Otherwise, to inhibit Akt Ser/Thr kinase, cells were serum-starved for 4 hours and incubated with 200 nM wortmannin in Dimethyl-Sulfoxide (DMSO) for 30 minutes at 37°C. Controls were carried out in similar conditions in the absence of λ phosphatase or in DMSO without wortmannin.

### Co-immunoprecipitation

Transfected HEK293 cells were washed in ice-cold PBS and lysed in ice-cold JS buffer (50 mM Hepes, 150 mM NaCl, 1% Glycerol, 1% Triton-X100, 1.5 mM MgCl_2_, 5 mM EGTA, 1 mM PMSF, 10 μg/ml Leupeptin, 10 μg/ml Aprotinin, 1 μM NaVO_4_). Cellular extracts were incubated with anti-FLAG M2 affinity gel (SIGMA-Aldrich) or with anti-HA antibody for 2 hours at 4°C. After incubation the affinity resins were washed three times in NET buffer (50 mM Tris-HCl pH 7.4, 150 mM NaCl, 0.1% Triton X-100, 1 mM EDTA, 1 mM PMSF, 10 μg/ml Leupeptin, 10 μg/ml Aprotinin, 1 mM NaVO_4_) and the adsorbed proteins analyzed by electrophoresis on a 10% polyacrylamide gel, transferred onto nitrocellulose membranes and revealed with specific antibodies.

### Immunofluorescence

HeLa cells were grown on glass cover slips, serum-starved for 4 hours and incubated with 100 ng/ml of EGF for 20 minutes at 37°C and 5% CO_2_. Cells were cooled on ice, washed three times in ice-cold PBS, then fixed in 4% paraformaldehyde (SIGMA-Aldrich) for 30 minutes at room temperature and washed in 0.1 M glycine in PBS. Cells were made permeable with 0.1% Triton X-100 for 5 minutes and incubated in blocking solution (10% FBS in PBS) for 40 minutes. HeLa cells were incubated over-night at 4°C with primary antibodies diluted in blocking solution, washed three times in PBS and incubated with secondary antibodies coupled to different fluorochromes (Alexa Fluor 488-coupled anti-mouse from Molecular Probes and Rhodamine Red-X-coupled anti-rabbit from Jackson Immuno-Research) for 1 hour at 37°C.

After washing in PBS, coverslips were mounted in Prolong Antifade (Molecular Probes) and images were acquired on a confocal microscope (Leica TCS SP2) or on Olympus IX 70 with Nanomover^® ^and softWoRx DeltaVision (Applied Precision) with a U-PLAN-APO 100× objective.

For assaying endocytosis, HeLa cells were grown on coverslips and transiently transfected with various cDNAs. After 24 hours from transfection cells were serum-starved for 4 hours, then incubated with TRITC-EGF (2 μg/ml) or TRITC-Transferrin (100 μg/ml) (Molecular Probes) for 20 minutes at 37°C and 5% CO_2_. Internalization of the fluorescence signal was monitored as described above. Nuclei were stained with Hoechst 33258 or 4,6-diamidino-2-phenylindole dihydrochloride (DAPI).

### Spot synthesis

Cellulose membrane-bound peptides were automatically synthesized according to standard SPOT synthesis protocols [27] using a Spot synthesizer MultiPep Spotter (Intavis AG, Germany). To limit background signals, all cysteines were replaced by serines. The generated arrays of peptides were synthesized on Amino-PEG500-UC540 Sheet (acid hardened) (Intavis AG, Germany). The membrane, was activated in Ethanol (3 times for 10 minutes), washed 3 times in PBS, blocked in PBS/BSA 5% and then incubated with Glutathione S-Transferase (GST) fusion proteins (10 micrograms/ml) in PBS/BSA 5% Incubation with GST alone at the same concentration in the same buffer is used as negative control. The membrane was then treated with anti GST antibody and finally with peroxidase labeled anti goat antibody. Chemiluminescent signals were acquired with a LAS3000 instrument (FujiFilm) and analyzed with the software AIDA.

## Authors' contributions

LT carried out the characterization of the 14-3-3 interactions, AC performed the initial experiments and the characterization of the SH3 dependent interactions, ES and MC performed the localization and co-immunoprecipitations, SC performed the signaling experiments, GC and LC participated in the design of the study and LC drafted the manuscript.

## Supplementary Material

Additional file 1 **Co-immunoprecipitation of POB1 with Grb2 and 14-3-3 at endogenous level is not EGF dependent**. HeLa cells were starved for 4 hours in serum deprived medium and induced by addition of EGF at 100 ng/ml, when indicated. Cells were lysed and treated as described. Protein lysates were immunoprecipitated with anti-POB1 (lane 2,3) and mock antibody in lane 1. The co-immunoprecipitated proteins were separated on SDS-PAGE, transfered onto nitrocellulose membranes and probed with anti-Grb2 or anti-14-3-3 zeta. Input is 5% of the amount loaded in the lanes of co-immunoprecipitations. Click here for file

Additional file 2 **POB1 localization in different sub cellular compartments: Localization is not EGF dependent**. HeLa cells were stained with anti-POB1 antibodies together with antibodies for markers of specific cellular structures: anti-clathrin CHC (staining coated pits), anti-EEA1 (early endosomes), anti-CD63 (late endosomes), anti-GM130 (Golgi), and anti-LAMP2 (lysosomes) Secondary antibodies were: Alexa-Fluor 488-coupled anti mouse, to stain the cell structure markers (green) and Rhodamine-conjugated anti rabbit, to stain POB1 (red). Images were acquired on Olympus IX 70 with Nanomover^® ^and softWoRx DeltaVision. In "merge", a yellow color indicates co localization. In panel A, HeLa were not treated with EGF. In panel B HeLa were treated with EGF 100 ng/ml. Click here for file

Additional file 3 **POB1 scanning: SH3 binding**. This file provides additional data to Fig. 3A. Scanning of the POB1 proline-rich from Pro308 to Val365, with the SH3 domain of Amphihphysin II (No 2-38), Grb2 carboxy-terminal SH3 (No. 39-76) and GST (No.77-114) as negative control. The sequence of the peptides is reported. Signal intensity of binding is reported in column labeled QL, as arbitrary units. Best binders are shown in red. Click here for file

Additional file 4 **POB1 338–352 Alanine scanning**. Additional data to Fig. 3B. Alanine Scanning Mutagenesis of the region POB1 338-PPTPPPRPQKTHSRA-352. The mutagenized peptides are indicated in the first column. The signal intensity obtained by probing with each SH3 domain or the GST alone is reported in second column. The intensity belonging to mutagenized peptides that have lost binding are in red. Click here for file

Additional file 5 **POB1 scanning: 14-3-3 binding**. Additional data to Fig. 5A. The first three spot positions are positive control peptides. From spot No. 25 to No. 188 (row 2–8) are the POB1 peptides obtained by scanning the POB1 sequence from L181 to L521. From spot No. 193 to No. 228 are the peptides containing a phosphorylated serine or threonine in the eighth position, as indicated by a s/t. From No. 229 to No. 264 are the same peptides as above but bearing an unphosphorylated Ser or Threo, indicated by S/T. From No. 265 to No. 280 are controls. The peptides were challenged with 14-3-3 sigma fused to GST. Intensity is reported in arbitrary units. Click here for file

Additional file 6 **POB1 increase affects EGF-dependent phosphorylation of Erk1–2 and Shc**. Fig 1. HEK293 were transfected with a vector encoding the full lenght protein POB1 isoform2, fused to the Myc epitope or the control of the empty vector. As a control, HEK293 were transfected with a vector encoding the Dynamin K44A fused to the Green Fluorescent protein (GFP). After 24 hours, cells were starved for 16 hours in serum deprived medium and induced by addition of Epidermal Growth factor (EGF) at 100 ng/ml final concentration. After 1 or 5 minutes (as indicated), 1 mg. of the cell lysate was immunoprecipitated with anti-phosphotyrosine antibody 4G10, while 50 micrograms of the lysate was loaded to visualize the input. Protein lysate were separated on SDS-PAGE, transfered onto nitrocellulose membranes and probed with anti-Shc antibody (panel a) or anti-Erk_1–2 _antibody (panel b). Cell extracts were normalized probing with anti-tubulin antibody. The immunoprecipitations were normalized by probing with anti-IgG light chain (25 kDa). Transfection efficiency is shown (panel c), by probing with anti-Myc antibody (for POB1) or anti-GFP antibody (for Dynamin K44A). Fig. 2. Band intensity from the experiment shown in the previous figure is acquired and measured by means of the AIDA program (Raytest). The phosphorylated proteins immunoprecipitated were normalized by comparison with the IgG (left) or to the unmodified counterpart: The figures on the right are the ratio between the tyrosin-phosphorylated and the total protein without EGF induction and after 1 or 5 minutes of EGF induction. The input protein was normalized by comparison with the tubulin. Fig. 3. Cellule HEK293 were transfected with a vector encoding the central portion (308–365) of the protein POB1 (PRD1) fused to the Green Fluorescent protein (GFP) and the empty vector, encoding GFP, as a negative control. After 24 hours, cells were starved for 16 hours in serum deprived medium and induced by addition of Epidermal Growth factor (EGF) at 100 ng/ml final concentration. After 1 or 5 minutes (as indicated), 1 mg. of the lysate was immunoprecipitated with anti-phosphotyrosine antibody 4G10, while 50 micrograms of the lysate was loaded to visualize the input. Protein lysate were separated on SDS-PAGE, transfered onto nitrocellulose membranes and probed with anti-SHC antibody (panel a) or anti-Erk_1–2 _antibody (panel b). Transfection efficiency is shown (panel c), by probing with anti-GFP antibody. Cell extracts were normalized probing with anti-tubulin antibody. The immunoprecipitations were normalized by probing with anti-IgG light chain (25 kDa). Fig. 4. Band intensity from the experiment in previous figure was acquired and measured by means of the AIDA program (Raytest). The figures on the right are the ratio between the tyrosin-phosphorylated and the total protein without EGF induction and after 1 or 5 minutes. Phosphorylated and input proteins were normalized. Fig. 5. **EGF induction control**. Cellule HEK293 Phoenix, Hela and A431 were serum starved for 16 hours (lane 1,3,5) and stimulated by EGF (100 ng/ml) addition (lane 2,4,6). Proteins were separated on SDS-PAGE and probed with anti-phosphotyrosine antibody 4G10. Click here for file
