# Supplementary figures and images for: The central proline rich region of POB1/REPS2 plays a regulatory role in epidermal growth factor receptor endocytosis by binding to 14-3-3 and SH3 domain-containing proteins
Source: BMC Biochem. 2008 Jul 22;9:21. doi: 10.1186/1471-2091-9-21 (PMC2494995; doi:10.1186/1471-2091-9-21)

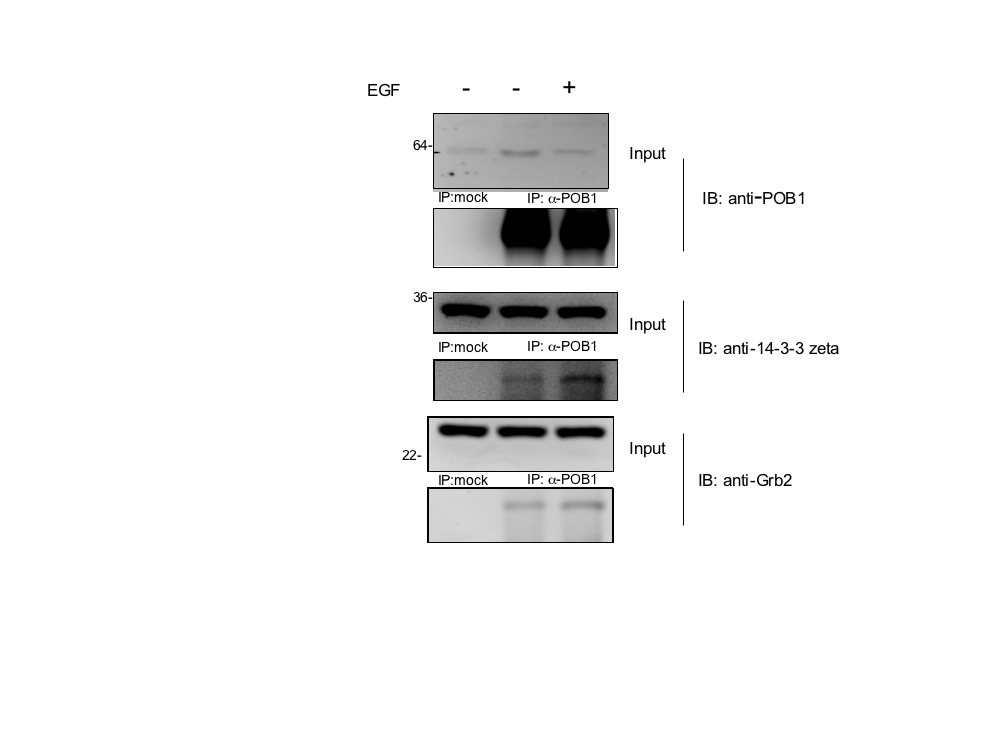

Supplement: Additional file 1 — Co-immunoprecipitation of POB1 with Grb2 and 14-3-3 at endogenous level is not EGF dependent. HeLa cells were starved for 4 hours in serum deprived medium and induced by addition of EGF at 100 ng/ml, when indicated. Cells were lysed and treated as described. Protein lysates were immunoprecipitated with anti-POB1 (lane 2,3) and mock antibody in lane 1. The co-immunoprecipitated proteins were separated on SDS-PAGE, transfered onto nitrocellulose membranes and probed with anti-Grb2 or anti-14-3-3 zeta. Input is 5% of the amount loaded in the lanes of co-immunoprecipitations. [file 1471-2091-9-21-S1.tiff]

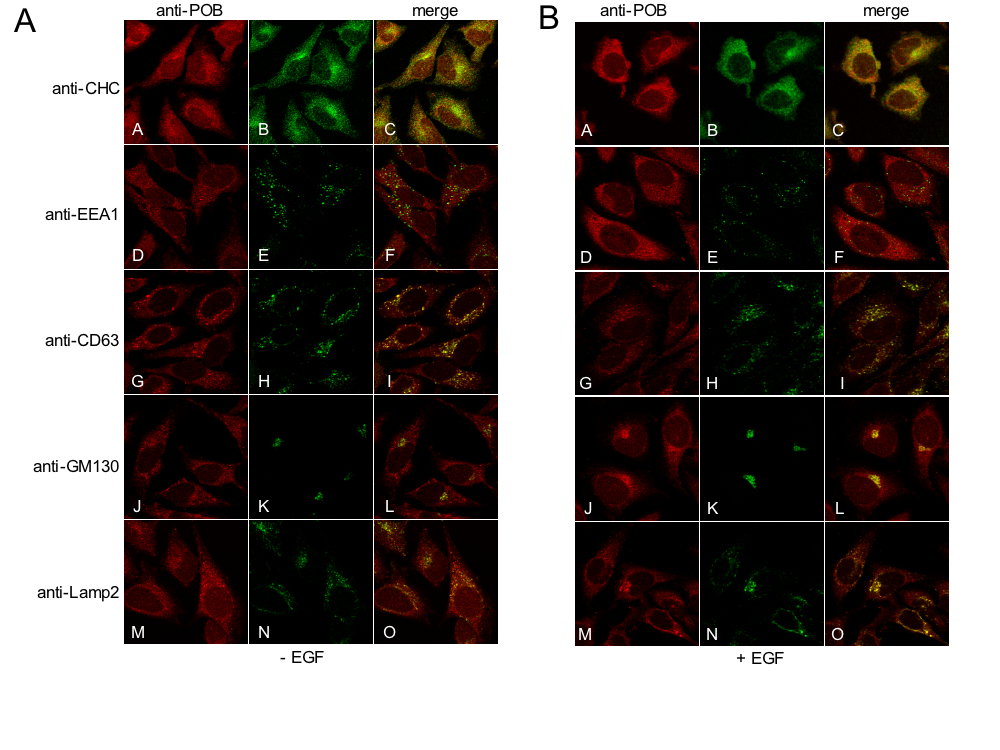

Supplement: Additional file 2 — POB1 localization in different sub cellular compartments: Localization is not EGF dependent. HeLa cells were stained with anti-POB1 antibodies together with antibodies for markers of specific cellular structures: anti-clathrin CHC (staining coated pits), anti-EEA1 (early endosomes), anti-CD63 (late endosomes), anti-GM130 (Golgi), and anti-LAMP2 (lysosomes) Secondary antibodies were: Alexa-Fluor 488-coupled anti mouse, to stain the cell structure markers (green) and Rhodamine-conjugated anti rabbit, to stain POB1 (red). Images were acquired on Olympus IX 70 with Nanomover® and softWoRx DeltaVision. In "merge", a yellow color indicates co localization. In panel A, HeLa were not treated with EGF. In panel B HeLa were treated with EGF 100 ng/ml. [file 1471-2091-9-21-S2.tiff]
