# Supplementary material for: The central proline rich region of POB1/REPS2 plays a regulatory role in epidermal growth factor receptor endocytosis by binding to 14-3-3 and SH3 domain-containing proteins
Source: BMC Biochem. 2008 Jul 22;9:21. doi: 10.1186/1471-2091-9-21 (PMC2494995; doi:10.1186/1471-2091-9-21)
Supplement: Additional file 6 — POB1 increase affects EGF-dependent phosphorylation of Erk1–2 and Shc. Fig 1. HEK293 were transfected with a vector encoding the full lenght protein POB1 isoform2, fused to the Myc epitope or the control of the empty vector. As a control, HEK293 were transfected with a vector encoding the Dynamin K44A fused to the Green Fluorescent protein (GFP). After 24 hours, cells were starved for 16 hours in serum deprived medium and induced by addition of Epidermal Growth factor (EGF) at 100 ng/ml final concentration. After 1 or 5 minutes (as indicated), 1 mg. of the cell lysate was immunoprecipitated with anti-phosphotyrosine antibody 4G10, while 50 micrograms of the lysate was loaded to visualize the input. Protein lysate were separated on SDS-PAGE, transfered onto nitrocellulose membranes and probed with anti-Shc antibody (panel a) or anti-Erk1–2 antibody (panel b). Cell extracts were normalized probing with anti-tubulin antibody. The immunoprecipitations were normalized by probing with anti-IgG light chain (25 kDa). Transfection efficiency is shown (panel c), by probing with anti-Myc antibody (for POB1) or anti-GFP antibody (for Dynamin K44A). Fig. 2. Band intensity from the experiment shown in the previous figure is acquired and measured by means of the AIDA program (Raytest). The phosphorylated proteins immunoprecipitated were normalized by comparison with the IgG (left) or to the unmodified counterpart: The figures on the right are the ratio between the tyrosin-phosphorylated and the total protein without EGF induction and after 1 or 5 minutes of EGF induction. The input protein was normalized by comparison with the tubulin. Fig. 3. Cellule HEK293 were transfected with a vector encoding the central portion (308–365) of the protein POB1 (PRD1) fused to the Green Fluorescent protein (GFP) and the empty vector, encoding GFP, as a negative control. After 24 hours, cells were starved for 16 hours in serum deprived medium and induced by addition of Epidermal Growth factor (EG [file 1471-2091-9-21-S6.doc]

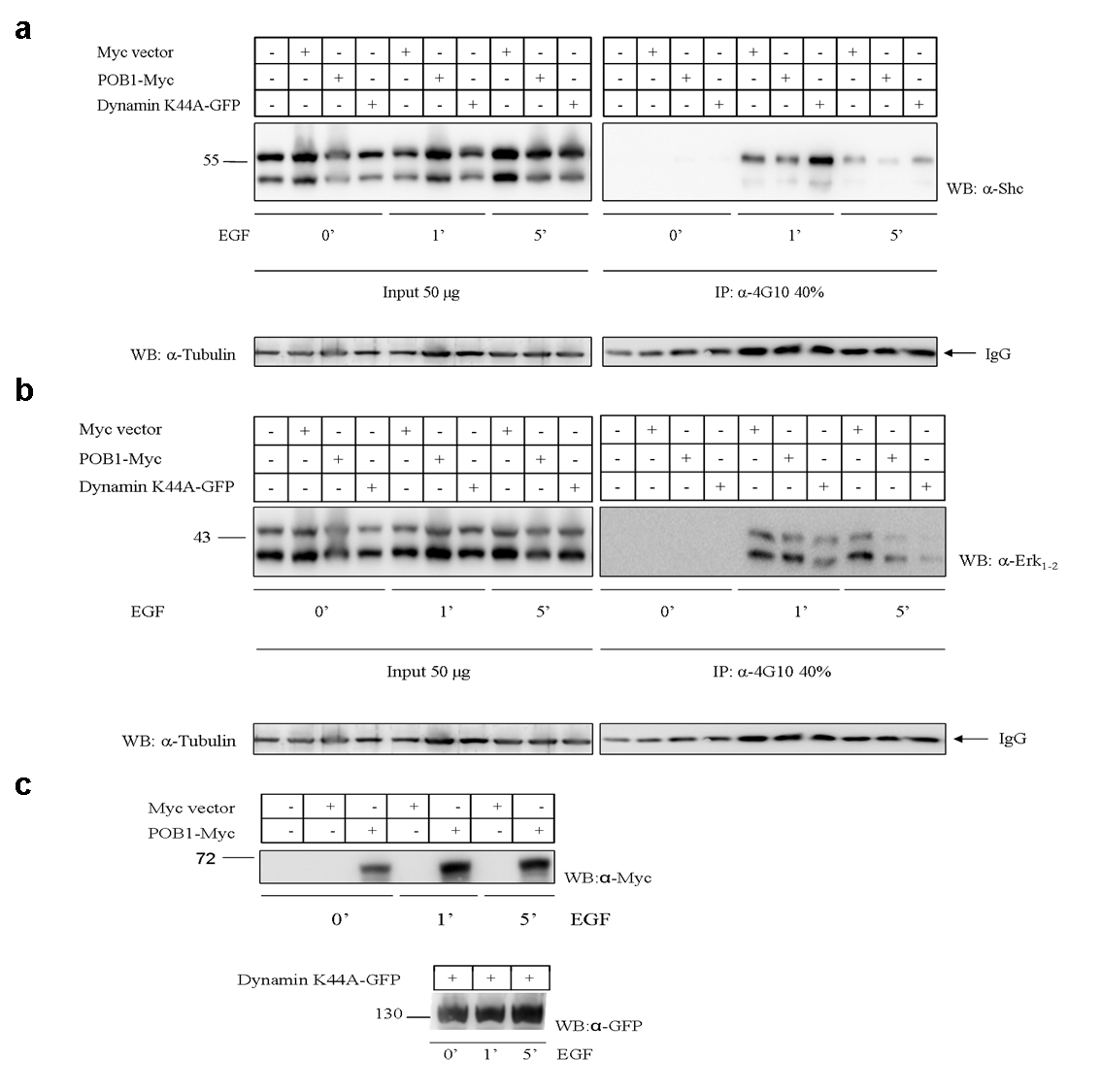


Additional file 6- Fig 1.


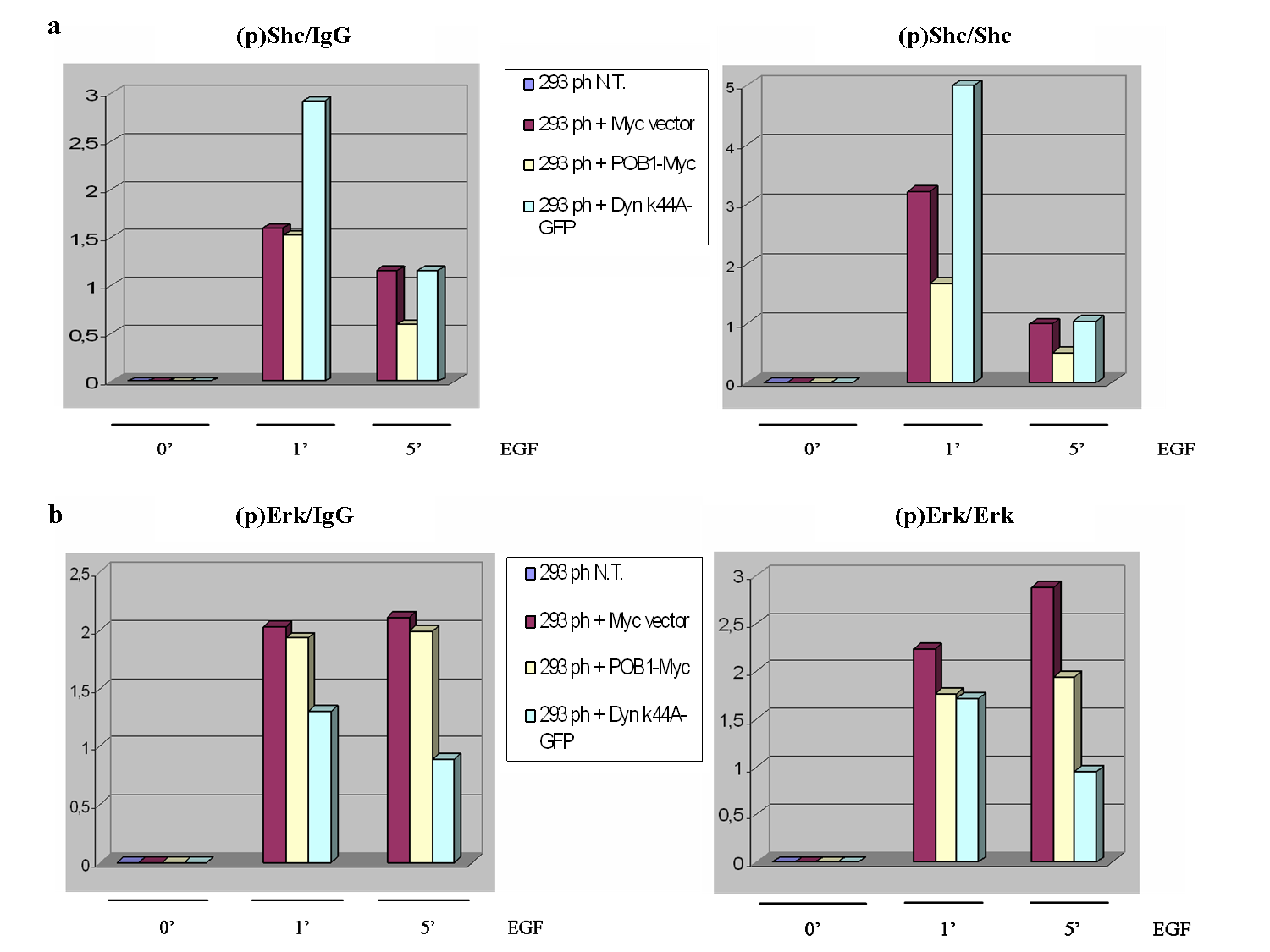


Additional file 6- Fig. 2.


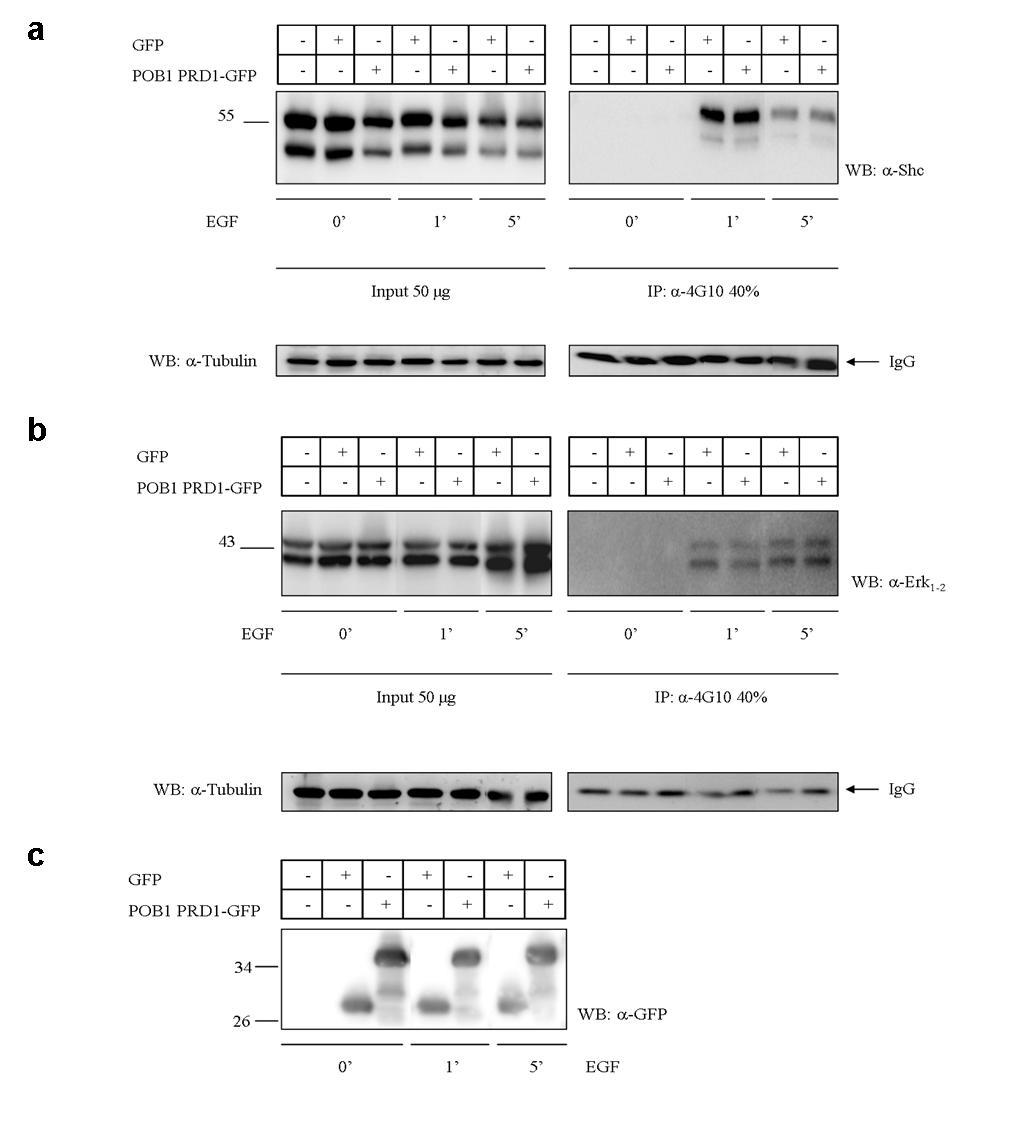


Additional file6- Fig. 3.


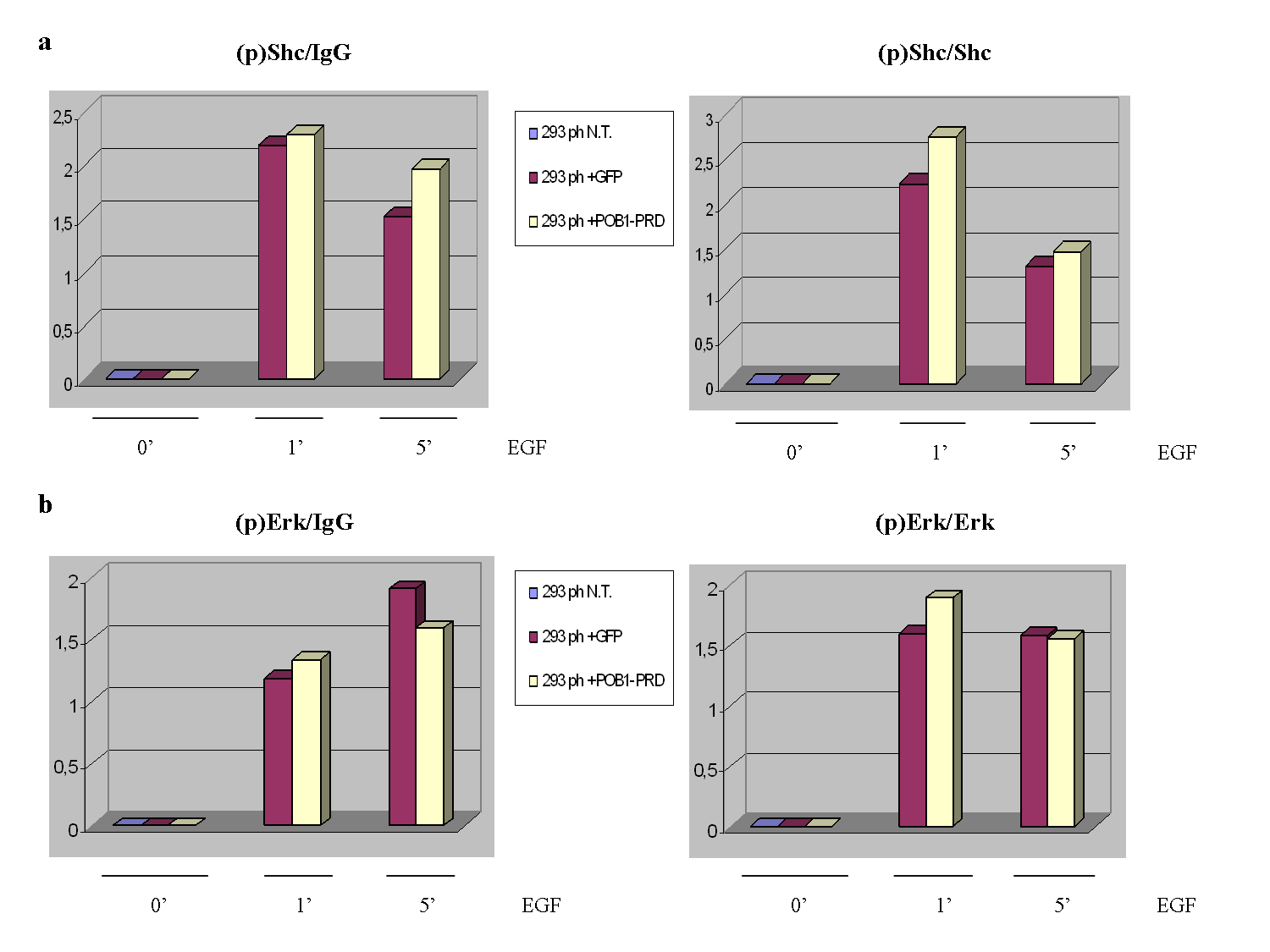


Additional file6- Fig. 4.


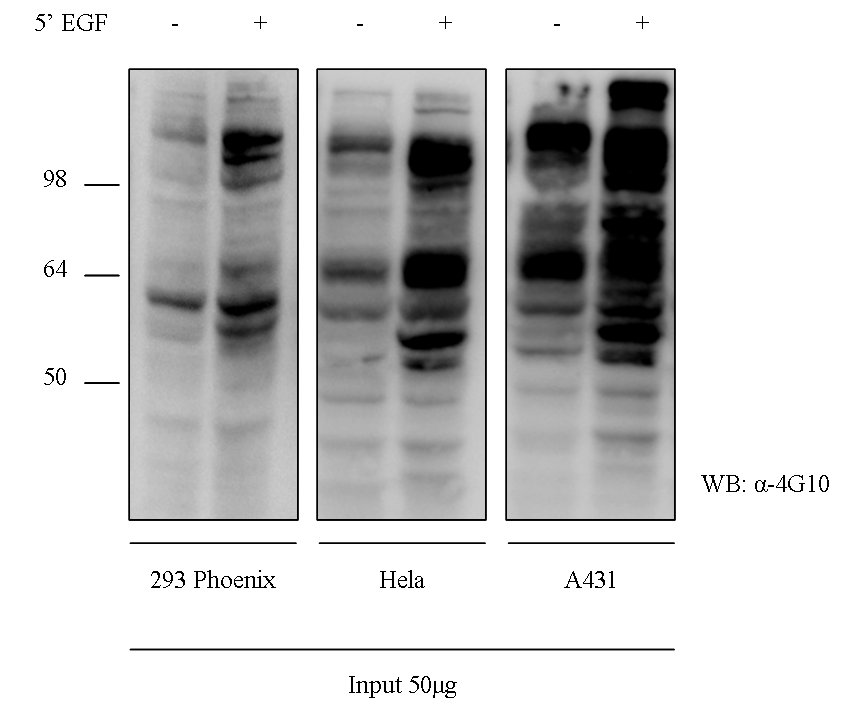


Additional file6- Fig. 5.
